# Unveiling the Occupational Exposure to Microbial Contamination in Conservation–Restoration Settings

**DOI:** 10.3390/microorganisms10081595

**Published:** 2022-08-08

**Authors:** Carla Viegas, Renata Cervantes, Marta Dias, Bianca Gomes, Pedro Pena, Elisabete Carolino, Magdalena Twarużek, Robert Kosicki, Ewelina Soszczyńska, Susana Viegas, Liliana Aranha Caetano, Ana Catarina Pinheiro

**Affiliations:** 1 H&TRC—Health & Technology Research Center, ESTeSL—Escola Superior de Tecnologia da Saúde de Lisboa, Instituto Politgy Research Center, 1990-096 Lisbon, Portugal; 2 Public Health Research Centre, NOVA National School of Public Health, Universidade NOVA de Lisboa, 1099-085 Lisboa, Portugal; 3 Comprehensive Health Research Center (CHRC), NOVA Medical School, Universidade NOVA de Lisboa, 1169-056 Lisboa, Portugal; 4 Department of Physiology and Toxicology, Institute of Experimental Biology, Faculty of Natural Sciences, Kazimierz Wielki University, Chodkiewicza 30, 85-064 Bydgoszcz, Poland; 5 Research Institute for Medicines (iMed.uLisboa), Faculty of Pharmacy, University of Lisbon, 1649-003 Lisbon, Portugal; 6 HERCULES Laboratory—Cultural Heritage Protection Studies, University of Evora, Palácio do Vimioso, Largo Marquês de Marialva, 8, 7000-809 Evora, Portugal

**Keywords:** occupational exposure assessment, *Aspergillus*, azole resistance, mycotoxins, cytotoxicity

## Abstract

Assuring a proper environment for the fulfillment of professional activities is one of the Sustainable Development Goals and is contemplated in the One Health approach assumed by the World Health Organization. This particular study is applied to an often neglected sector of our society—the conservators/restorers—despite the many health issues reported by these professionals. Three different specialties (textiles, paintings and wood sculpture) and locations were selected for evaluation by placement of electrostatic dust cloths. After treatment of the samples, bacterial and fungal contamination were assessed, as well as mycotoxin determination, the presence of azole-resistant strains and cytotoxicity of the microorganisms encountered. Bacteria were only present in one of medias used and showed relatively low numbers. The highest level of contamination by fungi was identified in one of the textiles settings. The textile area also showed the highest variability for fungi. *Aspergillus* sp. are one indicator of possible environmental issues, and *A.* sections *Fumigati* and *Circumdati* were particularly relevant in two of the settings and identified in all of them. No mycotoxins were detected and the large majority of the fungi identified were non-cytotoxic. Overall, these can be considered low-contaminated environments but attention should be given to the *Aspergillus* sp. contamination. Additional studies are needed not only to make these results more robust, but also to test if the environmental sampling alone is the best approach in a setting where there is very little movement and dust displacement and where professionals are in very close proximity to the artefacts being treated, which may suggest the existence of a micro-atmosphere worth evaluating and comparing to the obtained results.

## 1. Introduction

Among all microorganisms, fungi cause the degradation of cultural heritage sites to the greatest extent [1]. In the case of archives, some of the fungi present in paper documents, surfaces and air from archives, libraries and museums are also a threat to human health [2]. Due to their enormous enzymatic activity and their ability to grow at low water activity levels values, fungi are able to inhabit and to decay paintings, textiles, paper, parchment, leather, oil, casein, glue and other materials used for historical art objects. In museums and their storage rooms, climate control, regular cleaning and microbiological monitoring are essential in order to prevent fungal contamination.

It is mandatory for Portuguese employers to assess and prevent occupational exposure to chemical, physical and biological risks, as in all European countries [3]. Several studies have reported that exposure to microorganisms, such as bacteria and fungi, can originate respiratory diseases [4,5,6]. Nevertheless, those of biological origin are less recognized and reported than chemicals [7].

Conservators–restorers—professionals that handle priceless cultural heritage artefacts maintaining them for future generations—can work in a myriad of settings. From small private ateliers to large, state-run facilities, handling organic substrates, such as paintings or books or, conversely, working with stone or metals, doing their job high on a scaffold or bent over a textile. Whatever the location, position or material being handled, the exposure to the different hazards carried by each specialty should be addressed.

The biological hazards are, presumably, more relevant in the settings where the conservator–restorer handles organic substrates. Anyone who has ever entered an archive or library can recall a particular scent, and part of this aroma comes from paper, rag, leather or parchment degradation [8,9]. In addition, some of these deterioration issues come from the activity of microorganisms, acting on the organic substrate. Handling potentially contaminated cultural heritage artefacts may provoke a diverse array of health effects on the staff, due to the increased exposure [2,10,11,12]. In fact, high microbiological contamination, previously reported in museums, libraries and archives, may be harmful to workers [13,14,15].

Conservation measures and treatments used to inhibit fungal growth in paper-based items of cultural heritage include mechanical, chemical and biological methods, such as gamma rays and ethylene oxide fumigation [16]. If some of these disinfecting measures, which are intended to fragment fungal DNA, present suboptimal efficacy, they might contribute for the development of fungal tolerance, which is a risk factor for the development of fungal resistance in the long-term. Fungal resistance to medical azoles has been described in recent years as an important public health concern, which is expected to increase in the next years due to the current scenario of climatic changes [17,18,19].

Additionally, fungi are known to produce mycotoxins, their exometabolites that can be toxic for humans and animals. Mycotoxins are produced by specific fungal genera, mainly by *Aspergillus, Penicillium, Alternaria, Fusarium* and *Claviceps* [20,21]. Several mycotoxins are carcinogenic or probably carcinogenic to humans, as evaluated by the International Agency for Research on Cancer [22]. Mycotoxins are considered the most frequently occurring natural contaminants in the diet of humans and animals. Due to climate change, an increased magnitude and/or frequency in the exposure of humans to mycotoxins is expected to occur in temperate regions of Europe [23]. Mycotoxins can resist to adverse environmental factors, such as high or low temperatures, and can persist long after the death and disintegration of the fungal species responsible for their production [24]. Therefore, and due to the health effects related with exposure to mycotoxins when studying fungal contamination is reasonable to also study mycotoxins contamination [25].

This study aimed to assess microbial contamination present in four different work settings: textiles (2), paintings and sculpture (mostly wood based). The study was performed in the Lisbon area, applying electrostatic dust cloths as sampling method. The screening of azole-resistance profile, *Aspergillus* sections detection, as well as the mycotoxins and cytotoxicity assessment was also performed. Education and close collaboration of mycologists and restorers are needed to develop object specific methods for the conservation and treatment of contaminated objects.

## 2. Materials and Methods

### 2.1. Working Settings Assessed 

**Figure 1:**
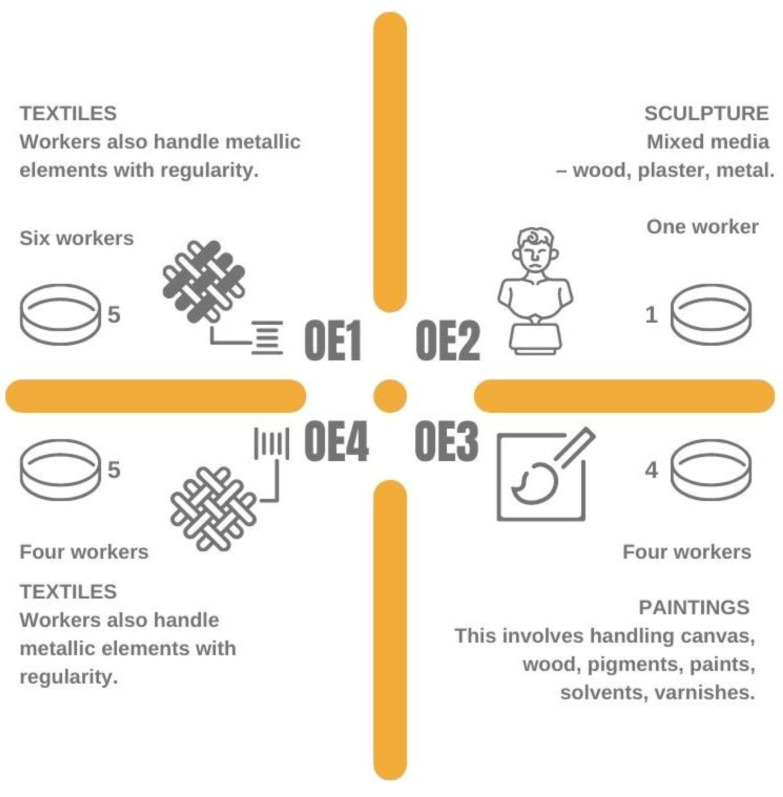
Sampling locations, OE1 and OE4 for textiles, OE2 for sculpture and OE3 for the painting’s restoration studio.

This study sampling campaign was conducted between May and June 2021 in four locations, three in the city of Lisbon and another one in a smaller coastal town. This last one was a private home-based studio, where a single conservator handles historic and artistic objects that can be made from a variety of substrates, some organic, some inorganic and most a composition of both. The several objects in line to be treated are kept at this location and the work is seldom initiated and maintained in different objects at the same time to make up for the obligatory intervals some treatments demand. The other private studio is dedicated to the restoration of paintings. At the time of the evaluation it was being used by three conservators and a master’s student. It is a relatively small studio, with two different stories and uses natural ventilation only. As happened in the previous case, there is also an accumulation of other paintings besides the ones that are being treated at the moment, either because the finalized work has not been collected, is between treatment phases or has not been initiated yet. The remaining two settings analyzed are both public run studios and both tend to the restoration of textiles. These are larger rooms and the only pieces that are on display are being treated by the conservators. At the time of the evaluation, one of the studios had 6 workers (this number can shift to 4 in case there is a low demand for conservation work) and the other had 4 workers in the premises (Figure 1).

### 2.2. Sampling Approach Characterization through Culture-Dependent Methods

**Figure 2:**
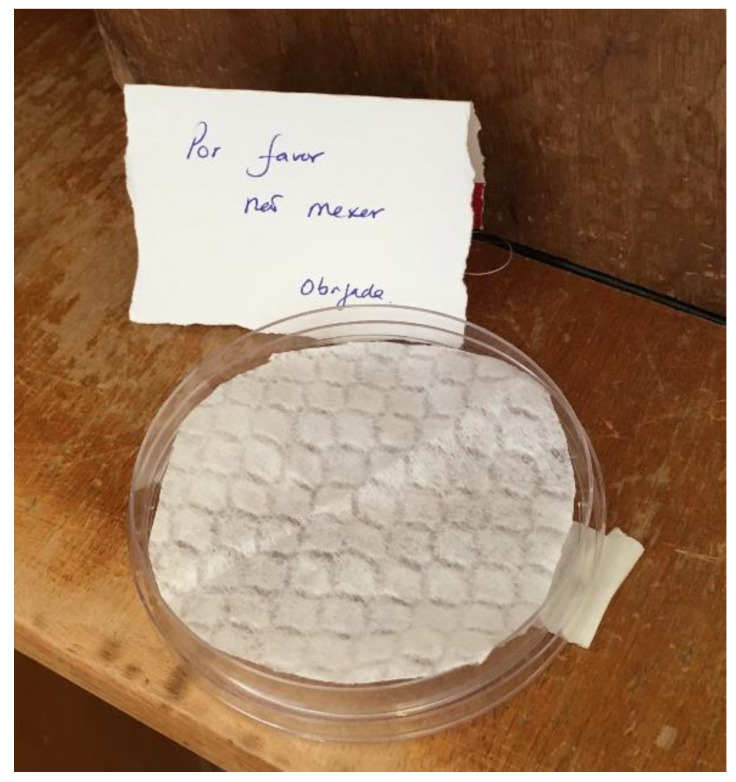
Example of one of the EDCs placed at the assessed locations. A request for non-disturbance accompanies the EDC.

The EDCs were placed in each sampling site (Figure 2) for 30 days and transported under refrigeration (0–4 °C) to the laboratory for further analyses [26]. EDCs were weighted and processed with 20 mL of 0.1% Tween 80 saline (0.9% NaCl). For fungal assessment malt extract agar (MEA) supplemented with chloramphenicol (0.05%), and dichloran-glycerol agar (DG18) were used. EDC samples were incubated at 27 °C for 5–7 days. For bacteria assessment, tryptic soy agar (TSA, 30 °C, 7 days) and violet-red bile agar (VRBA 35 °C, 7 days) were used for mesophilic bacteria and coliforms (Gram negative bacteria), respectively. Microbial contamination densities (colony-forming units, CFU·g^−1^, CFU·m^−2^, CFU·m^−2^·day^−1^) were calculated as previously reported [27,28]. Fungal species were preliminary identified microscopically following procedures previous published [29]. Negative controls were employed to ensure the inexistence of background contamination, namely culture media (all samples) and extracts of control samples (EDC) without prior use were submitted to the same assays.

### 2.3. Azole Resistance Screenin

Sabouraud dextrose agar (SDA) (Frilabo, Maia, Portugal), either alone or supplemented with 4 µg/mL itraconazole (ITZ), 2 µg/mL voriconazole (VCZ), or 0.5 µg/mL posaconazole (PSZ), were used to screen fungal resistance to medical azoles (adapted from [30,31]). The *A. fumigatus* ATCC 204305 reference strain, and a pan-azole-resistant *A. fumigatus* were used as controls (both strains provided by National Health Institute Doutor Ricardo Jorge, IP). Briefly, SDA media plates inoculated with samples’ extracts from all the EDC were incubated at 27 °C (to enable optimal conditions for fungal growth) for three to four days. After incubation, fungal colonies were counted and identified by microscopy, as previously described [32].

### 2.4. Molecular Detection of Aspergillus Sections

The extracts (8.8 mL) from the EDCs were used for molecular detection of *Aspergillus* sections [26]. Fungal DNA was extracted using the ZR Fungal/Bacterial DNA MiniPrep Kit (Zymo Research, Irvine, CA, USA) and molecular identification was performed by Real Time PCR (qPCR) using the CFX-Connect PCR System (Bio-Rad, Amadora, Portugal). Reactions included 1× iQ Supermix (Bio-Rad, Amadora, Portugal), 0.5 μM of each primer, and 0.375 μM of TaqMan probe in a total volume of 20 μL. Amplification followed a three-step PCR: 40 cycles with denaturation at 95 °C for 30 s, annealing at 52 °C for 30 s, and extension at 72 °C for 30 s.

A non-template control and a positive control consisting of DNA obtained from a reference that belonged to the culture collection of the Reference Unit for Parasitic and Fungal Infections, Department of Infectious Diseases of the National Institute of Health, from Dr. Ricardo Jorge were used. These strains have been sequenced for ITS, B-tubulin, and Calmodulin.

### 2.5. Mycotoxins Analysis

Nineteen samples were screened for mycotoxins presence. In all samples, 38 mycotoxins were analyzed by HPL-MS (HPLC) Nexera (Shimadzu, Tokyo, Japan) with a mass spectrometry detector API 4000 (Sciex, Foster City, CA, USA) following the same laboratory procedures described in previous papers [27,28]. The mycotoxin concentration was calculated using external calibration. The limits of detection (LOD) obtained for each mycotoxin with the analytical method used are presented in Table 1.

### 2.6. Assessment of Cytotoxicity

The cell viability of human lung epithelial (A549), human liver carcinoma (HepG2) and swine kidney (SK) cells, exposed to EDC samples for 48 h at 5% CO_2_, 37 °C, and humid atmosphere, were determined by the 3-(4,5-dimethylthiazol-2-yl)-2,5-diphenyltetrazolium bromide (MTT) assay at 510 nm, as previously described [33]. Briefly, cells were at first maintained in Eagle’s minimum essential medium (MEM) supplemented with 10,000 units penicillin and 10 mg/mL streptomycin in 0.9% NaCl and fetal bovine serum (Sigma-Aldrich, St. Louis, MO, USA). After cell detachment (with 0.25% (*w/v*) Trypsin 0.53 mM EDTA), 100 µL cell suspension with densities of 2.0 × 10^5^ to 4.5 × 10^5^ cells/mL (Scepter™ 2.0 Cell Counter, Merck, NJ, USA) was transferred to a 96-well plate. Cells were then exposed to the EDC samples and cell viability was measured (ELISA LEDETECT 96, biomed Dr. Wieser GmbH; MikroWin 2013SC software). The threshold toxicity level was considered the lowest concentration dropping absorption to <50% of cell metabolic activity (IC50).

### 2.7. Statistical Analysis

Data were analyzed using SPSS statistical software for windows, version 27.0. The results were considered significant at the 5% significance level. To test the normality of the data, the Shapiro–Wilk test was used. For the comparison of bacterial contamination, fungal contamination and fungal resistance, the Kruskal–Wallis test was used, since the assumption of normality was not verified and given the small size of the sample. To study the relationship between bacterial contamination, fungal contamination and fungal resistance, Spearman’s correlation coefficient was used, since the assumption of normality was not verified. To assess species diversity, Simpson and Shannon indices, given by $\mathrm{Shannon}\;\mathrm{Index}\;\left(H\right)=-\sum_{i=1}^{s}p_{i}\ln\left(p_{i}\right)$ and $\mathrm{Simpson}\;\mathrm{Index}\;\left(D\right)=\frac{1}{\sum_{i=1}^{s}p_{i}^{2}}$, were used, where *p_i_* is the proportion (*n_i_*/*n*) of individuals of one particular species found (*n_i_*) divided by the total number of individuals found (*n*).

## 3. Results

### 3.1. Viable Bacterial Contamination

**Figure 3:**
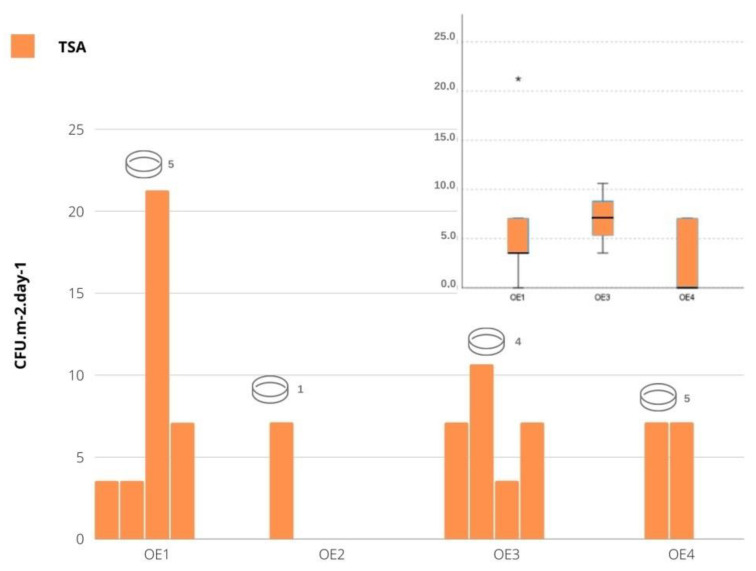
Bacterial contamination in the four sampled locations observed in TSA. On the top right is the boxplot for comparison of bacterial contamination in TSA medium between sampled areas (excluding OE2, since it has a single record). * severe outlier.

Total bacteria contamination ranged from 0 to 21.23 CFU·m^−2^ in one of the textile’s working area (OE1) and from 0 to 7.08 CFU·m^−2^ in the other one (OE4). It ranged from 3.54 to 10.62 CFU·m^−2^ in the paintings area (OE3) and in OE2 the counts were 7.08 CFU·m^−2^. Among the sampled areas, no statistically significant differences were detected ($\chi_{K-W}^{2}\left(2\right)=2.498,\;\;p=0.287$), with the sculpture area excluded, since it only had one observation. From the analysis of Figure 3 (boxplot), it can be seen that OE3 displays the highest contamination in TSA and the OE1 is the one presenting higher variability. No gram-negative bacteria (VRBA) were detected in any of the areas sampled.

### 3.2. Viable Fungal Contamination

**Figure 4:**
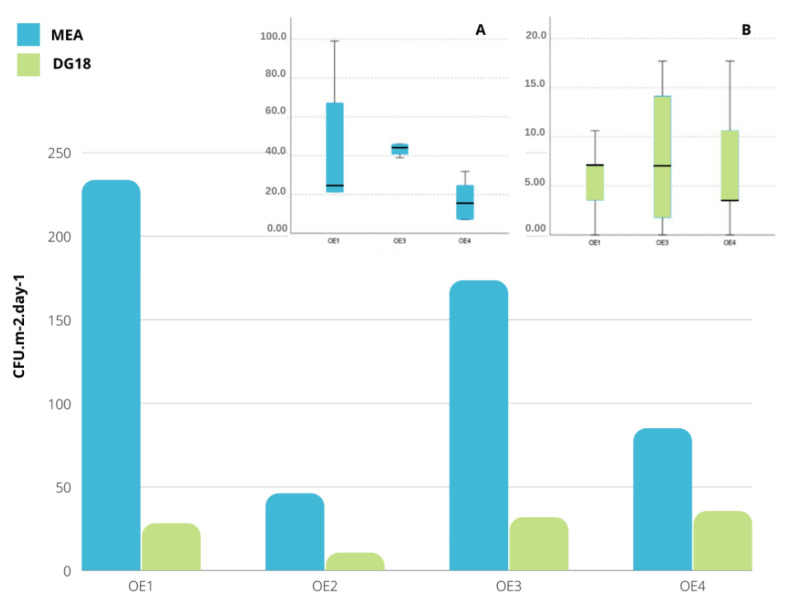
Total fungal counts on MEA and DG18 in samples from different areas (CFU·m^−2^·day^−1^). Boxplot for comparison of fungal contamination in MEA (**A**) and DG18 (**B**) media between sampled areas (excluding the sculpture area (OE2), since it has a single record).

Total fungal contamination in indoor sites was 233.5 CFU·m^−2^·day^−1^ on MEA and 28.3 CFU·m^−2^·day^−1^ on DG18 in the OE1 (textiles); 46 CFU·m^−2^·day^−1^ on MEA and 10.6 CFU·m^−2^·day^−1^ on DG18 in OE2 (sculpture); 173 CFU·m^−2^·day^−1^ in MEA and 31.8 CFU·m^−2^·day^−1^ in DG18 in the paintings area (OE3). The highest fungal counts were found in the OE1 textiles area (233.5 CFU·m^−2^·day^−1^ on MEA; 28.3 CFU·m^−2^·day^−1^ on DG18) (Figure 4).

In both MEA and DG18 medium, no statistically significant differences were detected between the sampled areas ($\chi_{K-W}^{2}\left(2\right)\;$ = 5.696, *p* = 0.058 and $\chi_{K-W}^{2}\left(2\right)\;$ = 0.177, *p* = 0.915, respectively). However, from the analysis of Figure 4 (boxplot), it can be seen that on MEA, OE3 was the one that presented the greatest fungal contamination and OE1 the one with the greatest variability. In DG18, one can see that OE1 and OE3 were the ones with the highest contamination, with the OE3 showing greater variability (sculpture area excluded as before).

**Figure 5:**
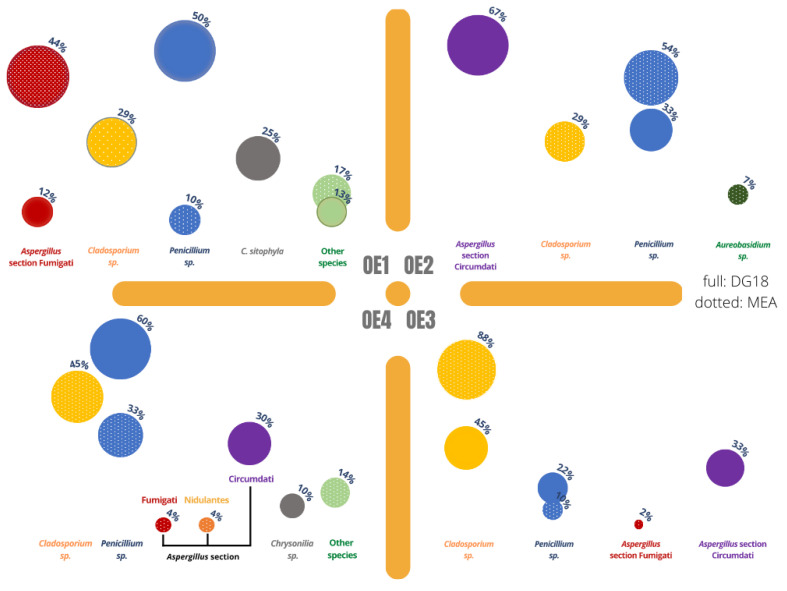
Fungal qualitative distribution in both MEA (dotted circles) and DG18 (full circles). Assigned to each circle is the percentage each genus occupies within the total results.

Concerning fungal distribution per sampling location, Figure 4 presents the quantitative results and Figure 5 presents the qualitative results.

The highest number of fungal species was obtained on the OE1 (8 species MEA; 4 species DG18, more details on all identified genera are on Table 2), closely followed by OE4 (6 species MEA; 3 species DG18). *Aspergillus* section *Fumigati* was the most common species obtained in OE1 in MEA (43.94%) while *Penicillium* sp. was the most common species obtained on DG18 (50%) in this same location; in the sculpture area—OE2—the most common species were *Aspergillus* section *Circumdati* on DG18 (66.66%) and *Penicillium* sp. on MEA (53.85%), and in OE3 (paintings) the most common species observed was *Cladosporium* sp. both in MEA (87.76%) and in DG18 (44.44%). In OE4 *Cladosporium* sp. accounted for 44.84% in MEA, while *Penicillium* sp. was the prevalent genera on DG18 (60%).

Regarding *Aspergillus* sp., they were present in all the assessed environments. The highest value obtained in MEA (43.94%) was found in OE1 and in OE2 in DG18 (67%). On MEA, the areas with the highest values of the genera were the OE1 (43.94%), followed by the OE4 (8.16%). OE3 displays a lower percentage in MEA (2.04%). The genus was not identified in the MEA media in OE2 but accounts for 67% of the CFUs identified in this location when using DG18.

**Figure 6:**
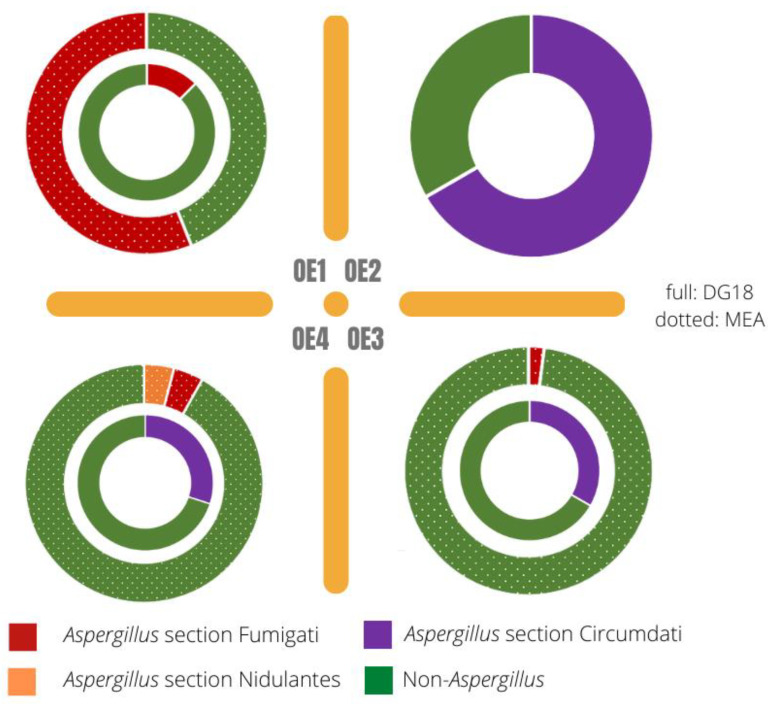
Aspergillus sections distribution by media and sampling place.

On DG18, two *Aspergillus* sections were identified, namely *Circumdati* (96.67%) and *Fumigati* (12.50%), also on MEA, two sections were reported, as follows: *Fumigati* (50.06%) and *Nidulantes* (4.08%). As for sections identification in OE1, one *Aspergillus* section was detected both on MEA (43.94% *Fumigati)* and DG18 (12.50% *Fumigati*). In OE2, no sections were detected on MEA and one section was detected on DG18 (66.66% *Circumdati).* In the painting area, OE3, two sections were identified, namely section *Fumigati* on MEA (2.04%) and section *Circumdati* on DG18 (33.33%). In the second textiles area, OE4, two sections were detected on MEA (4.08% *Fumigati* and 4.08% *Nidulantes)* and one section was detected on DG18 (30.00% *Circumdati)* (Figure 6).

Regarding species diversity on MEA, OE1 was the one with higher diversity (Shannon index (H) = 1.514, Simpson index (D) = 3.408) (Table 2).

### 3.3. Fungal Growth in Azole-Supplemented Media

**Figure 7:**
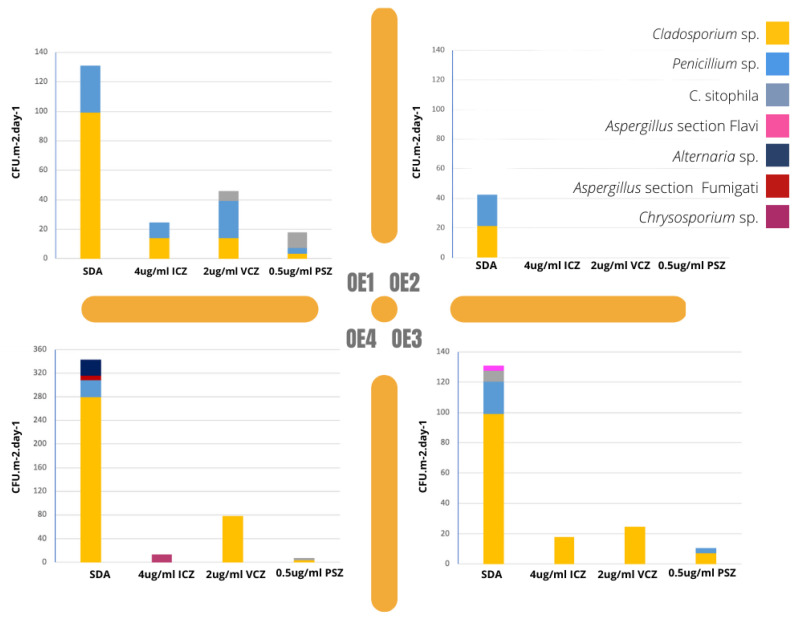
Fungal contamination (by azole screening method) per local (CFU·m^−2^·day^−1^). SDA = Saboraud dextrose agar; ICZ = itraconazole; VCZ = voriconazole; PSZ = posaconazole. Please mind the different scale used in OE4.

Regarding fungal contamination in azole-supplemented media, the results are presented in Figure 7. The most contaminated local was OE4 and the most frequent fungi in SDA (Saboraud Dextrose Agar) was *Cladosporium* sp. (5.0 × 10^2^ CFU·m^−2^·day^−1^), followed by *Penicillium* sp. (1.0 × 10^2^ CFU·m^−2^·day^−1^). Looking into each azole supplement individually, the most frequent fungi was *Cladosporium* sp. in voriconazole (VCZ) (7.8 × 10^1^ CFU·m^−2^·day^−1^) and itraconazole (ICZ) (3.2 × 10^1^ CFU·m^−2^·day^−1^), followed by *Penicillium* sp. in voriconazole (2.5 × 10^1^ CFU·m^−2^·day^−1^). *Aspergillus* sections *Flavi* (3.5 CFU·m^−2^·day^−1^) and *Fumigati* (7.0 CFU·m^−2^·day^−1^) were observed in SDA but not in any of the azole added media. The media with PSZ recorded the lowest contamination rate in all settings.

**Figure 8:**
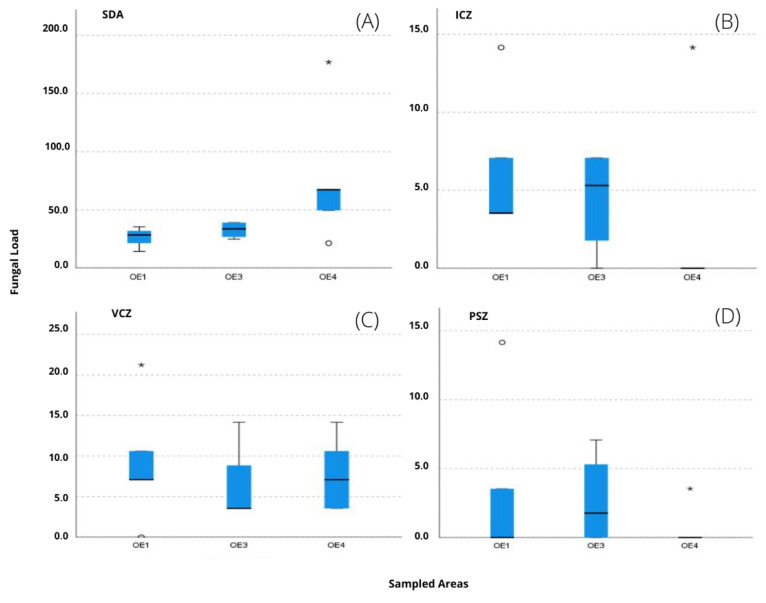
Boxplot for comparison of fungal load in SDA (**A**), ICZ (**B**), VCZ (**C**) and PSZ (**D**) between sampled areas (excluding the sculpture area (OE2), since it has a single record). Please note the different scales used. * severe outlier; ° moderate outlier.

Among the sampled areas, no statistically significant differences were detected in any of the media (p’s > 0.05) regarding fungal contamination. However, from the analysis of Figure 8A, it can be seen that, for the SDA, the textile area OE4 was the one with the highest values. The ICZ supplemented media (B) with the highest contamination was recorded in OE3. The textile working areas, both OE1 and OE4 presented similar results in in VCZ (C). Lastly, OE3 registered the highest contamination in PSZ (D).

### 3.4. Contamination of EDCs by Mycotoxins and Cytotoxicity Assessment

EDC sampling did not reveal the presence of any of the 38 mycotoxins evaluated at the sampled sites. Regarding the assessment of cell viability of the three distinct cell lines exposed to EDC, the results showed a majority of non-cytotoxic EDC, with only two samples exhibiting an IC50 value of 10 mm^2^/mL (one in A549 lung epithelial cells and another in SK cells).

### 3.5. Correlation Analysis

Only a significant positive correlation of moderate intensity was detected between bacterial contamination in TSA and fungal contamination in MEA (r_S_ = 0.621, *p* = 0.013), revealing that greater bacterial contamination in TSA is related to greater fungal contamination in MEA (Table 3).

## 4. Discussion

Conservators–restorers are a professional class that has yet to see some of its occupational hazards being correctly addressed [11,34,35,36]. It is not difficult to imagine experiencing allergic respiratory or dermatological symptoms when handling old documentation or textiles that have been exposed to dust or pesticides in the past [12,34,37,38]. As far as health issues are concerned, allergic symptoms (eye and throat pruritus, nasal congestion) and traumatic disorders from the adoption of awkward and stressful body positions, are the top cause of absenteeism or even the abandonment of the profession (personal inquiry). Performing a comprehensive microbiological analysis is, therefore, an essential step to understand the environments where these workers perform their activities. The assessed locations, as mentioned earlier, are mostly dedicated to the treatment of organic-based art pieces (in this case textiles, canvas and wood-based sculptures).

In what concerns fungal contamination assessment, different results were obtained with the two different culture media applied (MEA and DG18), following the trend also found in other occupational environments already assessed [27,39]. In fact, a greater number of fungal counts was obtained in MEA; what is expected since DG18 favors the presence of xerophilic fungi and restricts some fungi with fast growing rates, such as the Mucorales order [27,40]. There appears to be no correlation to be made between the type of materials being handled and the results obtained. The differences in results, for both bacterial and fungal contamination, can be related to the accumulation of dust and the movement due to the performed activities indoors [41,42,43] that may cause its displacement and deposition on the EDC. In addition, outdoor air flow, besides human activities, was reported to be the leading factor responsible for the fungal contamination indoors [44]. The two locations where the fungal counts were higher had other artworks that were not being handled at the moment, which adds to the dust deposition, although this was more evident in OE3. The fungal counts follow the same tendency as bacteria results, which also deem OE1 as the more contaminated. Because conservators spend long hours in the same position, devoted to one task, future studies might find it useful to include the collection of dust from each particular art piece for analysis, as well as the surrounding dust, as performed in this study.

In terms of variability—for fungi only—Figure 5 and Figure 6 confirm a higher variability in the two locations where textiles are handled. This variability is particularly noticeable in OE4, where three sections of *Aspergillus* were identified. Thus, the workstations OE1 and OE4 can be identified as hotspots for widespread *Aspergillus* and seen as a priority for risk management intervention. Previously, this genus was found to be the most prevalent on historical textiles and also reported that even with maintenance of recommended conditions, the growth of xerophilic fungi cannot be prevented [45]. In addition, several *Aspergillus* sections (*Circumdati, Flavi, Fumigati* and *Nidulantes*) considered as indicators of harmful fungal contamination were observed, indicating the need for the implementation of corrective measures [46,47]. For this particular case, and because it is organic materials that we are discussing, it is important also to address the impact these fungal and bacterial contaminations can have on the artwork itself. In fact, the textiles’ microbial colonization can promote conservators–restorers occupational exposure to these microbiologic risks but also the biodeterioration of historical textiles. Fungi can promote the biodeterioration of cellulosic and proteinaceous archaeological textiles, whereas bacteria are the main players for silk biodegradation [45,48]. Bacteria, however, do need higher water availability, and these art objects are normally kept within safe intervals of relative humidity and temperature [49].

The screening of azole resistance revealed one textile handling environment (OE4) as the one with the highest fungal load in Sabouraud media. Although a limited fungal diversity was found, with predominant *Cladosporium* and *Penicillium* sp., two important *Aspergillus* sections with toxigenic potential—*Flavi* and *Fumigati*—were present in the assessed environments. The results come to add another *Aspergillus* genus to the ones already identified (Figure 6). These results are in accordance with previous studies assessing textile specimens contaminated by fungi in Slovene and Jordanian museums, in which the dominant contaminant fungal species also belonged to the genus *Penicillium*, *Aspergillus* and *Cladosporium* [45].

The fact that *Cladosporium* sp. and *Penicillium* sp. were able to grow at tested concentrations of voriconazole and itraconazole must be further investigated in order to determine fungal susceptibility to other commonly used medical azoles for the treatment of fungal infections in humans. Moreover, although not determined during this assessment, azole-resistant *Fumigati* isolates have been increasingly reported in different environments [50] and described as a potential health menace, especially for immunocompromised individuals [18,51]. A deeper knowledge of fungal susceptibilities to azoles or other biocides is also relevant to guide the adoption of better fungal control strategies in restoration environments and suitable policies on cultural heritage conservation, while ensuring the maintenance of the effectiveness of antifungals in the treatment of infections in humans and animals [52,53].

As reported, none of the mycotoxins analyzed were detected. This might be related with many factors, such as the occupational environment characteristics (e.g., humidity, temperature, availability of fungal nutrients), and the materials being used and handled [54,55]. However, this does not mean that exposure might not happen in this occupational environment since the environmental conditions are constantly changing. It might also depend of the previous contamination of the materials and pieces to be handled and their storage conditions. Further studies are warranted to confirm these scenarios.

No relevant cytotoxicity was observed in EDC samples and the reduced number of samples does not allow further conclusions. Nevertheless, the use of relevant cell lines to assess biological effects and estimate health risks is a valid strategy for risk assessment [56,57,58].

## 5. Conclusions

This work presents important findings concerning microbial contamination in an occupational setting not commonly studied. In this particular study, the workplace environment where textiles were handled revealed itself as more prone to a diverse fungal contamination and, more specifically, to *Aspergillus* sp. contamination. The results, obtained with the techniques identified above, show low contaminated environments overall, considering and comparing with other settings. Thus, after the results are analyzed and compared with different studies using the same methodology, the behavior and particularities of these particular professionals and settings may warrant a conjugation of different approaches. Conservation and restoration is a task that is developed slowly and with care, and this means the workers do not engage in activities that contribute to the aerosolization of particles and, therefore, of fungi and bacteria. This is good news because it reduces the exposure to air contaminants, and good cleaning practices may render the working place safe. However, future studies must accommodate not only the environmental approach, with the EDC placed strategically on the workplace, but also the analysis of the painting, textile, etc., being treated because the conservator works in close proximity to the artefact and shares a micro-atmosphere with the piece itself. Comparing the EDC results with the results obtained by vacuum cleaning, the artefact will possibly increase our knowledge on the particularities of these settings.

As such, in future studies an innovative approach (One Health approach)—simultaneously targeting workplaces, workers (and users) and the cultural heritage—should be implemented to allow researchers to map the potential risk of microorganism’s dissemination and then, if needed, define an appropriate remediation strategy to simultaneously protect the health of workers and users and prevent further biodeterioration on the cultural heritage artefacts.

## Figures and Tables

**Table 1:** LOD values for the analyzed samples.

Mycotoxins	LOD
	(ng/g)
15-Acetyldeoxynivalenol	8
3-Acetyldeoxynivalenol Aflatoxin B_1_	4
Aflatoxin B_2_	1
Aflatoxin G_1_	1
Aflatoxin G_2_	1
Aflatoxin M_1_	1
Deepoxydeoxynivalenol	5
Deoxynivalenol	8
Deoxynivalenol-3-glucoside	5
Diacetoxyscirpenol	2
Fumonisin B_1_	4
Fumonisin B_2_	3
Fusarenon X	10
Griseofulvin	2
HT-2 toxin	4
Mevinolin	7
Monoacetoxyscirpenol	2
Mycophenolic acid	3
Neosolaniol	3
Nivalenol	4
Ochratoxin A	2
Ochratoxin B	2
Patulin	8
Roquefortine C	2
Sterigmatocystin	1
T-2 tetraol	2
T-2 toxin	2
T-2 triol	5
Zearalanone	2
Zearalenone	1
α-Zearalanol	2
α-Zearalenol	2
β-Zearalanol	2
β-Zearalenol	3

**Table 2:** Shannon and Simpson indexes to assess species diversity.

Sampled Areas	Species	Culture Media MEA (CFU/g^−1^·day^−1^)	Shannon Index (H)	Simpson Index (D)
OE1	*Alternaria* sp.	3.539	1.514	3.408
	*Aspergillus* section *Fumigati*	102.619		
	*Aureobasidium* sp.	10.616		
	*Chrysosporium* sp.	7.077		
	*Cladosporium* sp.	67.233		
	*Fusarium verticilloides*	7.077		
	*Penicillium* sp.	24.770		
	*Rhizopus* sp.	10.616		
Totals	8	233.546		
OE2	*Aureobasidium* sp.	3.539	0.227	1.199
	*Cladosporium* sp.	17.693		
	*Penicillium* sp.	24.770		
Totals	3	46.001		
OE3	*Aspergillus* section *Fumigati*	3.539	0.416	0.425
	*Cladosporium* sp.	152.159		
	*Penicillium* sp.	17.693		
Totals	3	173.390		
OE4	*Aspergillus* section *Fumigati*	3.539	1.977	0.371
	*Aspergillus* section *Nidulantes*	3.539		
	*Cladosporium* sp.	38.924		
	*Mucor* sp.	3.539		
	*Penicillium* sp.	28.309		
	*Trichoderma* sp.	7.077		
Totals	6	84.926		

**Table 3:** Study of the relationship between bacterial and fungal contamination and fungal resistance: Results of Spearman’s correlation coefficient.

		Bacteria	Fungi		Fungal Resistance			
		VRBA	MEA	DG18	SDA	ITZ	VCZ	PSZ
Bacteria	TSA	-	0.621 *	−0.003	0.191	−0.149	−0.403	0.480
	VRBA		-	-	-	-	-	-
Fungi	MEA			0.209	−0.329	−0.072	−0.177	0.239
	DG18				−0.016	0.139	−0.482	0.073
Fungal resistance	SDA					−0.269	−0.505	0.157
	ICZ						−0.098	0.018
	VCZ							−0.108

*. Correlation is significant at the 0.05 level (2-tailed).

## Data Availability

Through the UIDB/05608/2020 and UIDP/05608/2020.
